# Genetic Improvements in Rice Yield and Concomitant Increases in Radiation- and Nitrogen-Use Efficiency in Middle Reaches of Yangtze River

**DOI:** 10.1038/srep21049

**Published:** 2016-02-15

**Authors:** Guanglong Zhu, Shaobing Peng, Jianliang Huang, Kehui Cui, Lixiao Nie, Fei Wang

**Affiliations:** 1 National Key Laboratory of Crop Improvement, MOA Key Laboratory of Crop Ecophysiology and Farming System in the Middle Reaches of the Yangtze River, College of Plant Science and Technology, Huazhong Agricultural University, Wuhan, Hubei Province 430070, China

## Abstract

The yield potential of rice (*Oryza sativa* L.) has experienced two significant growth periods that coincide with the introduction of semi-dwarfism and the utilization of heterosis. In present study, we determined the annual increase in the grain yield of rice varieties grown from 1936 to 2005 in Middle Reaches of Yangtze River and examined the contributions of RUE (radiation-use efficiency, the conversion efficiency of pre-anthesis intercepted global radiation to biomass) and NUE (nitrogen-use efficiency, the ratio of grain yield to aboveground N accumulation) to these improvements. An examination of the 70-year period showed that the annual gains of 61.9 and 75.3 kg ha^**−**1^ in 2013 and 2014, respectively, corresponded to an annual increase of 1.18 and 1.16% in grain yields, respectively. The improvements in grain yield resulted from increases in the harvest index and biomass, and the sink size (spikelets per panicle) was significantly enlarged because of breeding for larger panicles. Improvements were observed in RUE and NUE through advancements in breeding. Moreover, both RUE and NUE were significantly correlated with the grain yield. Thus, our study suggests that genetic improvements in rice grain yield are associated with increased RUE and NUE.

Because of population growth, dietary shifts and biofuel consumption, the global food demand will double by 2050. The current rate of increased crop production will be insufficient to satisfy predicted demand^1^,^2^, and such problems will be further intensified by dwindling arable land area, environmental damage, climate change, and crop yield stagnation^2^,^3^,^4^,^5^. Therefore, to meet the global food demand, the yield potential of crops must be dramatically improved^6^.

Rice is the most important staple food crop in Asia and has significantly contributed to global food security in the past, and will continue to feed approximately half of the global population in the future^3^,^7^. The yield potential of irrigated rice has experienced two significant growth periods, with the first period driven by the introduction of semi-dwarfism and the second period driven by the utilization of heterosis^8^,^9^. Although multidisciplinary attempts have been made to increase the rice yield potential over the last decade, grain yield stagnation has been observed worldwide^4^,^10^,^11^,^12^,^13^. Therefore, understanding the physiological mechanisms underlying historical improvements in the rice yield potential would facilitate the identification of critical constraints for further improvements.

Over the last two decades, a number of studies have been performed to determine yield improvements that occurred through the process of breeding for wheat (*Triticum aestivum L.*)^14^, maize (*Zea mays L.*)^15^, rice (*Oryza sativa L.*)^16^, and soybean (*Glycine max Merr.*)^17^. In rice, annual grain yield gains of 75 to 81 kg ha^**−**1^ have been obtained for irrigated rice at International Rice Research Institute (IRRI) since 1966, gains of 42 kg ha^**−**1^ have been obtained for irrigated rice in Texas since 1944, and gains of 15.7 kg ha^**−**1^ have been obtained for upland rice in Brazil since 1984^16^,^18^,^19^. In China, genetic improvements have accounted for 62–74% of the yield increase in rice since the 1980s, with the remaining contributions induced by nitrogen fertilizer use and increased temperatures^20^,^21^. The harvest index and biomass production have played vital roles in the genetic improvement of rice before and after the 1980s, respectively, at IRRI^16^. In China, biomass production has been the main factor contributing to yield increases for indica hybrid rice since the late 1970s^22^, and sink size contributed significantly for japonica inbred rice in the northeast^23^. However, little is known of the physiological mechanisms underlying the genetic improvements in the grain yield of rice.

Radiation-use efficiency (RUE) is defined as the efficiency of using intercepted radiation to produce biomass by crops, and it is regarded as the only remaining major prospect for improving yield potential^24^. Recently, increasing attempts have been made to improve RUE by increasing the photosynthetic rate^6^,^12^,^25^,^26^,^27^. However, the literature on changes in RUE during genetic improvements to crops remains controversial. Early studies did not observe changes in RUE during the breeding process in wheat^28^,^29^, whereas more recent studies in wheat and soybean have demonstrated significantly improved of RUE in newer varieties^17^,^30^. Studies on the changes in RUE during genetic improvement in rice are still lacking. Nitrogen (N) is an essential element for plant growth and rice yield, which partially occurs through its influences on photosynthesis and RUE^31^. Genetic improvements in N uptake (N_UP_) and N use efficiency for grain production (NUEg) have been found in wheat^32^, maize^33^, and cotton^34^. In rice, a recent study found significantly higher N_UP_ and NUEg in super rice compared with older varieties in Jiangsu Province of China^35^. However, the relationship between NUE and RUE during the genetic improvement of rice grain yield has rarely been examined.

In this study, the grain yield, yield components, plant morphology, radiation interception, RUE, N_UP_ and NUEg were determined in a two-year field experiment using widely disseminated varieties in various decades since the 1930s in China. We determined the genetic improvements in the grain yield for rice varieties grown from 1936 to 2005 in Middle Reaches of Yangtze River, and found that significant increases in RUE and NUE contributed to the increased grain yields in the advance of rice breeding in Middle Reaches of Yangtze River.

## Results

### Climatic condition

**Figure 1:**
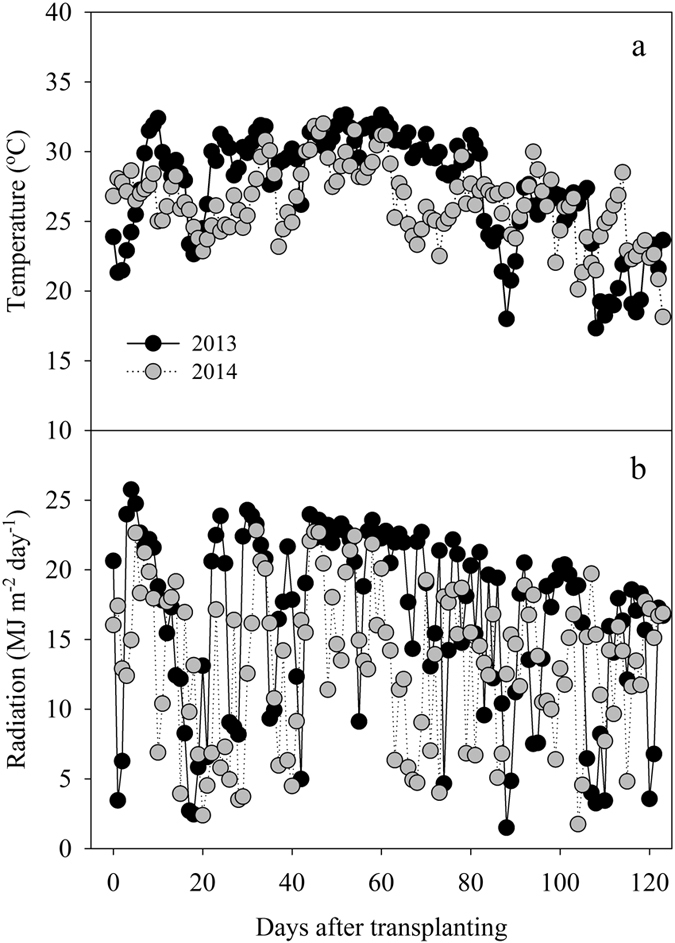
Daily mean temperature (**a**) and daily radiation (**b**) in 2013 and 2014.

The average daily maximum and minimum temperature during the growing season was 31.5 and 23.2 °C in 2013, respectively, and 30.1 and 22.5 °C in 2014, respectively. The mean daily solar radiation from May to October was 16.4 and 13.3 MJ m^**−**2^ day^**−**1^ in 2013 and 2014, respectively (Fig. 1). The average daily maximum and minimum temperature during the growing season was 1.4 and 0.7 °C higher in 2013 than in 2014, respectively. The mean daily solar radiation from May to October in 2013 was 23.3% higher than that in 2014 (Fig. 1).

### Genetic improvements in the grain yield and yield components

**Figure 2:**
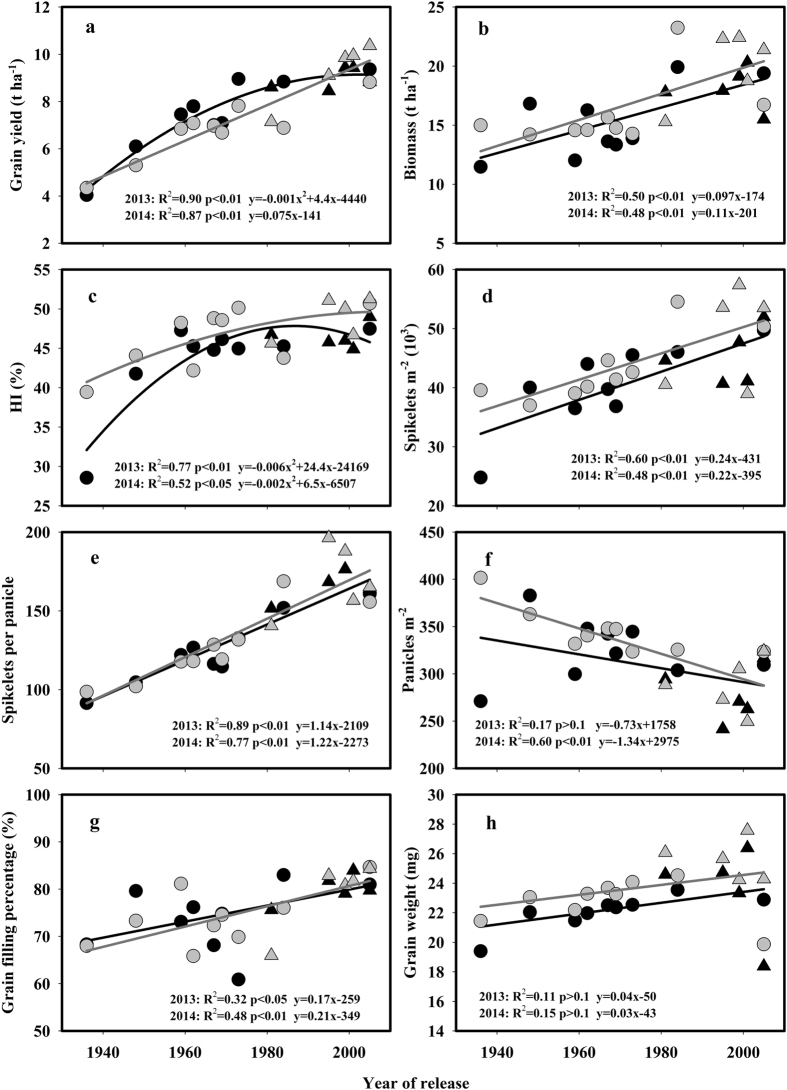
Changes in grain yield and yield components with the year of release of the rice varieties grown in 2013 (black symbols) and 2014 (gray symbols). Circles and triangles represent data for inbred and hybrid varieties, respectively.

Fourteen varieties that were released and widely cultivated from 1936 to 2005 in Middle Reaches of Yangtze River were assessed in this study (Table 1). The grain yield of newer varieties has been significantly increased compared with that of the older varieties. In 2013 and 2014, the grain yield increased to 61.9 and 75.3 kg ha^**−**1^ year^**−**1^, respectively, which corresponding to annual increases of 1.18 and 1.16%, respectively. Super-hybrid varieties released after 2000, such as YLY6 and YLY1, achieved grain yields close to 10 t ha^**−**1^, whereas the tall variety SLX produced a grain yield of 4.04 t ha^**−**1^ in 2013 and 4.33 t ha^**−**1^ in 2014 (Table 2). A quadratic relationship between the grain yield and the year of release was observed in 2013, which demonstrated a decreasing trend in the genetic improvements in the grain yield over the last two decades (Table 2; Fig. 2a). In 2014, the grain yield of varieties that had been released before 1990, with the exception of HHZ, the grain yield was higher. This trend resulted in a linear correlation between the grain yield and year of release in 2014 (Table 2; Fig. 2a). The genetic improvements in the grain yield since the 1930s have resulted from both an extended growth season and a higher daily grain yield (Fig. S1).

To dissect the causes of these improvements in grain yield, the yield components were measured (Table 2; Fig. 2). Biomass production was significantly increased according to the year of release after the 1980s, and these changes were driven by the utilization of heterosis (Fig. 2). A quadratic relationship between the harvest index and the year of release was observed and had an R^2^ value of 0.77 in 2013 and 0.52 in 2014. The harvest index of SLX, a tall variety, was 28.6 and 39.4% in 2013 and 2014, respectively, and these values were significantly lower than that of the semi-dwarf varieties (Table 3; Fig. 2c). Among the yield components, a significant increase in spikelets m^**−**2^ and grain filling percentage accounted for the genetic improvement in grain yield (Table 2; Fig. 2d,g). Newer varieties tended to present significantly fewer but larger panicles than the older varieties (Table 2 and Fig. 2e,f), and significant changes were not observed in grain weight during the breeding process (Table 2; Fig. 2h).

### Genetic improvements in pre-anthesis radiation use efficiency

The total incident radiation from transplanting to heading was increased along with the year of release because of the extended growing season (Fig. S2a). The total incident radiation from transplanting to heading was higher in 2013 than in 2014 because of different weather conditions between the two years (Table 3; Fig. 1). Lodging, which could potentially reduce radiation interception, occurred during grain filling period for some varieties, including SLX, GCA, GC2, SY63, TQ and YLY1 in 2013 and SLX, AZZ, ZZA, SY63, YLY6 and YLY1 in 2014. However, bamboos and ropes were immediately used to help lodging plants stand up so as to minimize the reduction in radiation interception and biomass production. Therefore, light interception was measured from transplanting to heading, and significant changes were not observed in the pre-anthesis radiation interception efficiency, although the leaf area index (LAI) significantly increased along with the advances in breeding (Table 3; Fig. S2b and S3a). Significant correlations (p < 0.1) were observed between the grain yield and intercepted radiation from transplanting to heading in 2013 and 2014 (Fig. S4c).

**Figure 3:**
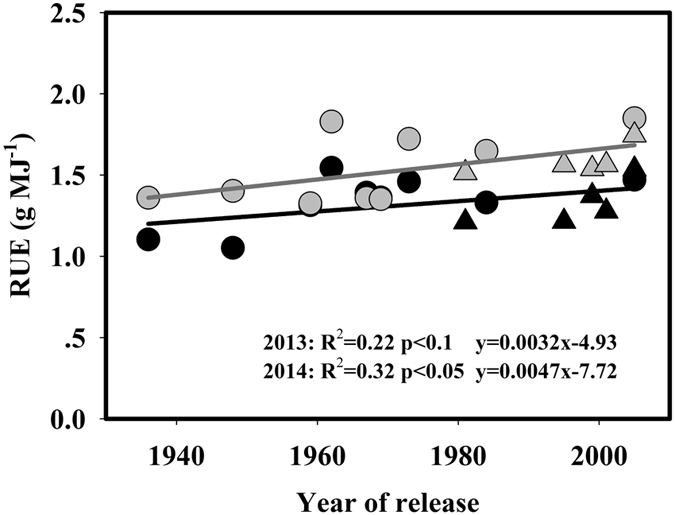
Changes in pre-anthesis RUE with the year of release of the rice varieties grown in 2013 (black symbols) and 2014 (gray symbols). Pre-anthesis RUE was calculated as the ratio of aboveground biomass at heading relative to the intercepted global radiation from transplanting to heading. Circles and triangles represent data for inbred and hybrid varieties, respectively.

The RUE for pre-anthesis intercepted global radiation (pre-anthesis RUE) was designated as the amount of biomass produced using intercepted global radiation from transplanting to heading, and the values ranged from 1.05 to 1.55 g MJ^**−**1^ in 2013 and from 1.35 to 1.85 g MJ^**−**1^ in 2014. Significant correlations (p < 0.1) between the pre-anthesis RUE and the year of release were observed in both 2013 (R^2^ = 0.22) and 2014 (R^2^ = 0.32). The genetic improvements in the pre-anthesis RUE were 0.0032 and 0.0047 g MJ^**−**1^ year^**−**1^ on an absolute basis, and 0.45 and 0.40% per year on a relative basis for 2013 and 2014, respectively. The AZZ and SLX varieties released in the 1930s and 1940s had the lowest pre-anthesis RUE, whereas the YLY1 and HHZ varieties released in the 2000s had the highest values (Fig. 3). Significant correlations were observed between the grain yield and pre-anthesis RUE at p < 0.1 in both 2013 and 2014 (Fig. S4d).

### Genetic improvement in nitrogen uptake and use efficiency

**Figure 4:**
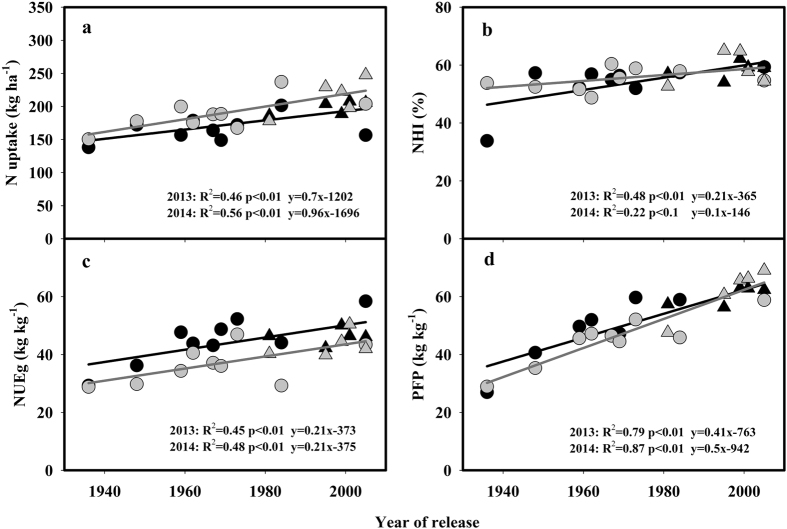
Changes in the nitrogen uptake, nitrogen harvest index, nitrogen use efficiency for grain production (NUEg), and partial factor productivity of applied nitrogen (PFP) with the year of release of the rice varieties grown in 2013 (black symbols) and 2014 (gray symbols). Circles and triangles represent data for inbred and hybrid varieties, respectively.

The total N uptake significantly increased (p < 0.01) along with the year of release, and the values ranged from 138 to 208 kg ha^**−**1^ in 2013 and from 150 to 248 kg ha^**−**1^ in 2014. In both years, the SLX variety released in the 1930s accumulated the lowest amount of N, whereas the YLY6 and YLY1 varieties, both released in the 2000s, had the highest N uptake (Table 4; Fig. 4a). The N harvest index (NHI) ranged from 33.9 to 62.2% in 2013 and from 48.8 to 65.0% in 2014. In 2013, the correlation between the NHI and the year of release was significant at p < 0.01 (R^2^ = 0.48), whereas in 2014, the correlation was significant at p < 0.1 (R^2^ = 0.22) (Fig. 4b).

The nitrogen use efficiency for grain production (NUEg) reflects the physiological efficiency of assimilated N for grain yield production, and this value was significantly increased along with the year of release (p < 0.01) (Fig. 4c). Genetic improvements in the NUEg were 0.21 kg kg^**−**1^ year^**−**1^ (0.84% per year) in 2013 and 0.20 kg kg^**−**1^ year^**−**1^ (0.57% per year) in 2014. The partial factor productivity of N fertilizer (PFP) reflects the use efficiency of applied N fertilizer in grain yield production, and the value was linearly correlated (p < 0.01) with the year of release with R^2^ of 0.79 in 2013 and 0.87 in 2014 (Fig. 4d). The PFP ranged from 26.9 kg kg^**−**1^ for SLX to 63.0 kg kg^**−**1^ for LYPJ in 2013 and from 28.9 kg kg^**−**1^ for SLX to 69.1 kg kg^**−**1^ for YLY1 in 2014 (Table 4). The genetic improvements in PFP were 0.41 and 0.50 kg kg^**−**1^ year^**−**1^ on an absolute basis, and 1.18 and 1.16% on a relative basis for 2013 and 2014, respectively. Both the total N uptake and NUEg significantly contributed to the increased grain yield at p < 0.01 in 2013 and 2014 (Fig. S5a,b).

## Discussion

### Genetic improvements in the grain yield

Taking the whole period of 70 years (from 1936 to 2005), annual gains of 61.9 and 75.3 kg ha^**−**1^ in grain yield were observed for rice varieties grown in Middle Reaches of Yangtze River in 2013 and 2014, respectively (Fig. 2a). The rate of genetic improvements in the grain yield showed in present study falls within the range reported by similar studies on rice at IRRI and in Texas^16^,^18^. However, the increasing trend was not linear in 2013 (Table 2; Fig. 2a), which is consistent with the recent yield stagnation observed in 79% of rice planting areas in China^4^. Despite the relatively lower rate of yield increases in the last two decades, breeding efforts have significantly improved the stability of grain yield for varieties since the 1990s. Compared with 2013, grain yield of varieties released prior to 1990s (except for SLX and NJ11) was reduced by 5.8–21.9% in 2014 due to the lower radiation and temperature, whereas grain yield of varieties released after 1990s (except for HHZ) was increased by 4.3–10.6% (Table 2). These results suggest that maintenance breeding has improved the adaptation of newer varieties to the environmental conditions of low radiation and low temperature that have a negative impact on older varieties^36^.

Grain yield is the product of biomass and harvest index^37^. Harvest index (HI) was increased significantly when the *sd1* gene was utilized in rice breeding in the 1950s^38^, and this improvement was demonstrated in this study by the significantly lower HI of the tall variety SLX (Table 2; Fig. 2c). Biomass has significantly increased since the end of the 1970s in present study due to the use of heterosis^13^. On the other hand, the grain yield could be divided into several components, namely spikelet m^**−**2^ comprised of panicles m^**−**2^ and spikelets panicle^**−**1^, grain filling percentage, and grain weight^37^. Among the yield components, the enlarged sink size caused by the heavier panicles largely accounted for the genetic improvements in grain yield of rice (Table 2; Fig. 2e), and this result was similar to the results found in an analysis of 21 indica hybrid varieties released since 1976^22^ and 12 indica inbred and hybrid varieties released since 1940s^35^ in China. A moderate number of tillers and large panicles have been the target traits in many rice breeding programs such as the new plant type (NPT) breeding program at IRRI^11^ and the “super” hybrid rice breeding program in China^39^. To maintain a high grain filling percentage for the large sink size, other morphological traits must be simultaneously improved to increase biomass production through a combination of the ideotype approach and the utilization of intersubspecific heterosis^11^. These traits are mostly related to the leaf morphology, such as the leaf length, angle and thickness and LAI of the top three leaves^39^. Modifications in these plant morphological traits result in the improvements in the light distribution and ventilation within the canopy, which could lead to increases in canopy photosynthesis^40^,^41^.

### Genetic improvements in RUE

From a physiological perspective, biomass at maturity is the product of total incident radiation during the rice growing period, efficiency with which radiation is intercepted by the crop (radiation interception efficiency), and efficiency with which intercepted radiation converted into biomass^42^. In the advance of breeding, the total incident radiation during the rice growth period has increased because the growing season has been extended (Fig. S1a and S2a).

RUE is one of the most promising traits for further improvements in the grain yield of rice^43^, since the efficiency of radiation interception has been significantly improved because plant stature and canopy architecture have been optimized^17^,^24^. A question remains as to whether RUE has been increased during the crop breeding process. Many lines of evidence indicate that improvements in RUE have contributed to genetic improvements in the grain yield of many crops, although some has argued that there has been little or no improvement in the conversion efficiency of radiation into biomass^6^,^17^,^30^. In present study, grain yield was significantly correlated with pre-anthesis biomass accumulation and pre-anthesis RUE, but not with post-anthesis biomass accumulation (Fig. S4a,b). RUE was significantly increased along with the year of release of the varieties, and a 27.8% improvement in pre-anthesis RUE was observed over the 70 years covered in present study (approximately 0.43% per year). Similar results have been reported in other crops. In soybean, the efficiency of light conversion to biomass has increased by approximately 36% over 84 years, and together with the improvement in light interception efficiency, these changes are responsible for the observed yield increases in the advance of breeding^17^. For wheat varieties developed from 1972 to 1995 in the UK, increases in pre-anthesis RUE drove increases in the number of grains and the accumulation of soluble carbohydrates for grain filling, which led to significant improvements in grain yield^30^. On one hand, the increase in RUE discussed above may have resulted from improvements in the intrinsic photosynthetic rate. In rice, the higher biomass of newer varieties released at IRRI has led to an increased light saturated photosynthetic rate per unit leaf area^44^. A similar trend has also been found in other studies in rice^45^,^46^. On the other hand, the optimization of plant architecture might have decreased the photoinhibition of the top leaves and increased the assimilation rate of lower leaves through an optimized distribution of radiation within the canopy^47^,^48^,^49^. Moreover, higher photosynthetic rates have been observed in rice during the grain filling period in newer varieties 45,46, and such changes could contribute to improvements of RUE during the grain filling period because the photosynthetic rate in the flag leaf after heading was positively correlated with grain yield^50^.

The pre-anthesis RUE in present study ranged from 1.05 to 1.85 g MJ^**−**1^ (Table 3), and these values are similar to that of two super-hybrid, two ordinary hybrid and two inbred rice varieties (1.08–1.66 g MJ^**−**1^) grown in Hunan and Guangdong provinces in China^51^, and to that of seven high-yielding rice varieties (1.29–1.72 g MJ^**−**1^) grown in Yunnan province of China and Kyoto of Japan^52^. Here, a significant difference was observed in pre-anthesis RUE values between 2013 and 2014 (Table 3). Significant variation of the RUE values in two consecutive experimental years was also found in soybean^14^, and this may have resulted from a significant negative relationship between RUE and intercepted (or incident) radiation^42^. Recently, a meta-analysis found that RUE was 18% higher in shaded conditions compared with that under full sunlight^53^.

### Genetic improvements in NUE

Several studies demonstrated that breeding for new varieties in rice significantly increased the response of grain yield to N applications^18^,^46^, which has led to an overuse of N fertilizers and a reduction in NUE in rice production^54^. In present study, we demonstrated that N uptake was significantly higher in newer varieties, and NUEg was also significantly increased (Fig. 4). These results are consistent with the results of a recent study in which yield improvements were accompanied by increases in N uptake and NUEg for varieties widely grown in Jiangsu Province over the past 70 years^35^. Improvements in N uptake and NUEg in the advance of breeding were also found in wheat^32^, maize^33^ and cotton^55^. These results indicate that the empirical viewpoint that newer varieties developed under conditions of ample N application have lower NUE is unauthentic, and show that the lower NUE frequently observed in rice production is mainly caused by inappropriate N management^35^,^54^.

In present study, both the increased N uptake and NUEg contributed to the increases in grain yield (Fig. S5a,b), because N significantly affects grain yield through its effect on both the source and sink. Increases in N uptake may have contributed to the improvement in RUE because RUE is dependent on photosynthesis and respiration^43^, and these metabolic processes are affected by plant N uptake^31^,^56^. On the other hand, larger N accumulation in vegetative and early reproductive growth stage is necessary for producing large number of spikelets^57^, and N top-dressing at the panicle initiation stage was most efficient in increasing spikelet number^58^. In present study, breeding for high yield is accompanied by a significant increase in the number of spikelets per panicle and N uptake and NUEg. Coincidences of QTLs for yield and its components with genes encoding cytosolic GS and the corresponding enzyme activity were detected in maize^59^ and rice^60^. Recently, genetic link between number of spikelets per panicle and nitrogen use efficiency in rice was demonstrated by one gene locus “*DEP1*”^61^,^62^. The gene is first cloned to reduce length of inflorescence internode, and increase number of grains per panicle and grain yield in rice^61^. Afterwards, a major rice NUE quantitative trait locus (*qNGR9*) is cloned, and interestingly this gene locus is synonymous with *DEP1*^62^.

## Conclusions

Yield potential has been the main target in rice breeding program under the pressure of increasing population. In this study, the grain yield was significantly increased with the year of release. The genetic improvements in the grain yield partially resulted from the significant increase in RUE. In addition, both nitrogen uptake capacity and nitrogen use efficiency for grain production in newer varieties was improved significantly, which contributed to the increase in grain yield. Currently, the world is facing challenges from environmental pollution, depletion of natural resources, climate change, and growing population, so crop yield potential has to be increased together with resource use efficiency. Compared with the theoretical maxima, there is still room for further improvement in RUE which is an important target in future breeding program. Recent progress in identification of NUE-related genes in rice may facilitate the breeding for high NUE. Overall, RUE and NUE should be concomitantly increased in the future.

## Materials and Methods

### Experiment design and plant materials

The experiments were conducted in a farmers’ field at Dajin Township (29°51′N, 115°53′E), Wuxue County, Hubei Province, China, during the rice-growing season from May to October in 2013 and 2014. The soil from experiment field had a texture of clay loam with pH 5.47, organic matter 29.10 g kg^−1^, total N 2.2 g kg^−1^, available P 12.14 mg kg^−1^ and available K 92.2 mg kg^−1^.

Fourteen historical indica mega varieties that were released from 1936 to 2005 were used in this study, and they were all cultivated as middle-season rice in a large-scale area at that time in the Middle Reaches of Yangtze River of China during the last 70 years. Among them, Shenglixian is a tall variety, Aizizhan, Guangchang’ai, Zhenzhu’ai, Nanjing11, Ezhong2, Guichao2, Teqing and Huanghuazhan are inbred rice, Shanyou63 is an ordinary hybrid rice, and IIYou725, Liangyoupeijiu, Yangliangyou6, Yliangyou1 are super high yielding rice varieties that were certified by China’s Ministry of Agriculture (Table 1).

All cultivars were arranged in a randomized complete block design with four replications and plot size of 5 × 6 m. Pre-germinated seeds were sown in seedbed. Seedlings (25 d old) were transplanted on 9 June in 2013 and 6 June in 2014. The planting density was 25 hills m^**−**2^ at a hill spacing of 30.0 cm × 13.3 cm with three seedlings per hill. Fertilizers included urea for N, single superphosphate for P and potassium chloride for K, and they were applied at the rates of 150 kg N ha^**−**1^, 40 kg P ha^**−**1^and 100 kg K ha^**−**1^. N fertilizer was split-applied at a ratio of 4:2:4 at basal (1 day before transplanting), tillering (7 days after transplanting), and panicle initiation. P and K were all applied at basal. The experimental field was flooded from transplanting until 7 days before maturity. Pests and weeds were intensively controlled using chemicals to avoid yield loss.

### Measurements

#### Plant sampling at heading

Plants from 12 hills in each plot were sampled at heading, and then separated into leaves, stems and panicles. The area of green leaf blades was measured with a Li-Cor area meter (Li-Cor Model 3100, Li-Cor Inc., Lincoln, NE, USA), and expressed as leaf area index (LAI). The dry weights of leaves, stems and panicles were measured after oven-drying at 70 °C to constant weight. The specific leaf weight was calculated as the ratio of leaf weight to leaf area. The aboveground total dry weight was the summation of dry weights in different plant parts.

#### Yield and yield components

At maturity, plants of 5 m^2^ in the center of each plot (to avoid border effect) were harvested to determine the grain yield which was adjusted to 14% moisture content. Grain moisture content was measured with a digital moisture tester (DMC-700, Seedburo, Chicago, IL, USA). Grain yield per day was calculated as the ratio of grain yield to total growth duration. Destructive sampling of 12 hills from each plot was conducted to determine the yield components. After the panicle number was counted, the plants were separated into straw and panicles. The straw dry weight was determined after oven drying at 80 °C to a constant weight. The panicles were hand threshed, and the filled spikelets were separated from the unfilled spikelets by submerging the spikelets in tap water, then half-filled and empty spikelets were separated by seed wind machine (FJ-1, China). Subsequently, three 30 g subsamples of filled spikelets, 15 g subsamples of half-filled spikelets, and 2 g subsamples of empty spikelets were collected to count the number of spikelets. The dry weights of the rachis and filled, half-filled and empty spikelets (unfilled spikelets) were determined after oven drying at 80 °C to a constant weight. The panicles m^**−**2^, spikelets per panicle, total spikelets m^**−**2^, 1000-grain weight, and harvest index were all calculated. Total dry weight (TDW) was calculated by summing the total dry matter of straw, rachis, filled and unfilled spikelets. Harvest index was calculated as the percentage of grain yield to the aboveground total biomass.

#### Radiation interception and use efficiency

The climate data (temperature and solar radiation) were collected from the weather station located within 2 km from the experimental site. A datalogger (CR800, Campbell Scientific Inc., Logan, Utah, USA) was used as the measurement and control module. A silicon pyranometer (LI-200, LI-COR Inc., Lincoln, NE, USA) and temperature/RH probe (HMP45C, Vaisala Inc., Helsinki, Finland) were used to measure total solar radiation and temperature, respectively. The total incident radiation were summation of daily global solar radiations from transplanting to maturity.

The canopy radiation interception (LI) was measured from transplant to heading in 2013 and 2014. LI was not measured during grain filling because of the occurrence of lodging. The measurements were performed between 1100 and 1300 h on clear-sky days at an interval of 7–15 days during the growing season with a line ceptometer (AccuPAR LP-80, Decagon Devices Inc., Pullman, WA, USA). In each plot, the light intensity inside the canopy was measured by placing the light bar in the middle of two rows and at approximately 5 cm above the water surface. The light intensity was then recorded above the canopy. In total, six measurements were performed in each plot, with three measurements performed in wider rows and three performed in narrower rows. The LI was calculated as the percentage of light intercepted by the canopy (light intensity above the canopy**-**light intensity below the canopy)/light intensity above the canopy^39^. The intercepted radiation during each growing period was calculated using the average LI and accumulated global radiation during the growing period. The intercepted global radiation from transplanting to heading (Ri) was the summation of intercepted global radiation during each growing periods as in Equation 1:


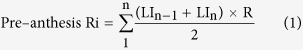


where n represents the time when LI was measured and R represents total global solar radiation during the period between two consecutive measurements of LI. LI at transplanting (LI_0_) was assumed to be zero.

Radiation use efficiency for intercepted global radiation from transplanting to heading (pre-anthesis RUE) was calculated as the ratio of aboveground total dry weight at heading relative to total intercepted global radiation based on Equation 2^63^.


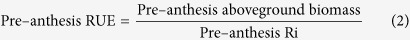


#### Nitrogen uptake and use efficiency

At maturity, after measurement of yield components and biomass from 12-hill samples, dry matter of each plant part [stem plus leaf (straw), filled grains, and unfilled grains plus rachis] was ground to powder to measure N concentration with Elementar vario MAX CNS/CN (Elementar Trading Co., Ltd, Germany). The total aboveground N uptake was then calculated as the product of the N concentration and dry weight of each aboveground part. Nitrogen harvest index (NHI) was calculated as the ratio of grain N content to total aboveground N uptake according to Equation 3. Nitrogen use efficiency for grain production (NUEg) was calculated as the ratio of grain yield over total aboveground N uptake according to Equation 4. Partial factor productivity of applied nitrogen fertilizer (PFP) was calculated as the ratio of grain yield to the fertilizer N input according to Equation 5.


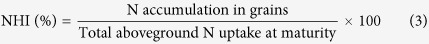



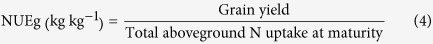



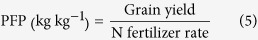


### Data analysis

An analysis of variance was performed with Statistix 8.0, and the mean values were compared based on the least significant difference (LSD) test at a 0.05 probability level. All of the figures were constructed and the regression analysis were performed using SigmaPlot 12.5.

## Additional Information

**How to cite this article**: Zhu, G. *et al.* Genetic Improvements in Rice Yield and Concomitant Increases in Radiation- and Nitrogen-Use Efficiency in Middle Reaches of Yangtze River. *Sci. Rep.* **6**, 21049; doi: 10.1038/srep21049 (2016).

## Supplementary Material

Supplementary Information

## Figures and Tables

**Table 1:** Information of rice varieties grown in different years since 1930s in Middle Reaches of Yangtze River.

Variety	Abbreviation	Year of release	Type	Province of release
Shenglixian	SLX	1936	inbred	Hunan
Aizizhan	AZZ	1948	inbred	Guangxi
Guangchang’ai	GCA	1959	inbred	Guangdong
Zhenzhu’ai	ZZA	1962	inbred	Guangdong
Nanjing11	NJ11	1967	inbred	Jiangsu
Ezhong2	EZ2	1969	inbred	Hubei
Guichao2	GC2	1973	inbred	Guangdong
Shanyou63	SY63	1981	hybrid	Fujian
Teqing	TQ	1984	inbred	Guangdong
IIyou725	IIY725	1995	superhybrid	Sichuan
Liangyoupeijiu	LYPJ	1999	superhybrid	Jiangsu
Yangliangyou6	YLY6	2001	superhybrid	Jiangsu
Huanghuazhan	HHZ	2005	inbred	Guangdong
Yliangyou1	YLY1	2005	superhybrid	Hunan

The information was from the website of China Rice Data Center (http://www.ricedata.cn/variety/varis/600877.htm).

**Table 2:** Grain yield and yield components of rice varieties grown in different years since 1930s in Middle Reaches of Yangtze River in 2013 and 2014.

Variety	Yield (t ha^−1^)	Panicles m^−2^	Spikelets per panicle	Spikelets m^−2^ (×10^3^)	Grain filling percentage (%)	Grain weight (mg)
2013						
SLX	4.04	271	91	24.8	68.3	19.4
AZZ	6.10	383	105	40.0	79.6	22.0
GCA	7.45	300	122	36.5	73.1	21.5
ZZA	7.80	348	127	44.0	76.2	22.0
NJ11	7.01	342	116	39.8	68.1	22.5
EZ2	7.09	322	115	36.8	74.8	22.4
GC2	8.95	345	133	45.5	60.9	22.5
SY63	8.61	294	152	44.6	75.6	24.6
TQ	8.84	304	152	46.0	83.0	23.5
IIY725	8.45	242	168	40.7	81.8	24.7
LYPJ	9.44	271	177	47.7	79.1	23.3
YLY6	9.43	263	157	41.1	84.0	26.4
HHZ	8.83	317	164	52.0	79.7	18.4
YLY1	9.36	309	161	49.7	81.0	22.9
Mean	7.96	308	139	42.1	76.1	22.6
LSD (0.05)	0.67	36.7	13	5.8	6.6	0.5
2014						
SLX	4.33	402	99	39.6	68.0	21.5
AZZ	5.30	363	102	37.0	73.3	23.1
GCA	6.88	332	118	39.1	81.1	22.2
ZZA	7.05	341	118	40.2	65.9	23.3
NJ11	7.00	348	129	44.6	72.4	23.7
EZ2	6.68	347	119	41.4	74.6	23.3
GC2	7.83	323	132	42.6	69.9	24.1
SY63	7.15	289	141	40.5	66.0	26.1
TQ	6.90	326	169	54.6	76.1	24.5
IIY725	9.10	273	196	53.6	82.9	25.7
LYPJ	9.85	305	188	57.4	80.8	24.2
YLY6	9.95	250	157	39.0	81.7	27.6
HHZ	8.85	324	156	50.3	84.7	19.9
YLY1	10.35	324	165	53.5	84.4	24.3
Mean	7.66	325	142	45.2	75.8	23.8
LSD (0.05)	0.54	25	10	3.7	4.4	0.36

**Table 3:** Total incident radiation from transplanting to heading, intercepted radiation from transplanting to heading, radiation interception efficiency from transplanting to heading, biomass, harvest index (HI) and pre-anthesis RUE of rice varieties grown in different years since 1930s in Middle Reaches of Yangtze River in 2013 and 2014.

Variety	Total incident radiation (MJ)		Intercepted radiation (MJ)		Radiation interception efficiency (%)		Biomass (g m^−2^)		HI (%)		RUE (g MJ^−1^)	
	2013	2014	2013	2014	2013	2014	2013	2014	2013	2014	2013	2014
SLX	911	855	708	636	77.8	74.4	1147	1499	28.6	39.4	1.10	1.38
AZZ	1161	869	911	609	78.6	70.1	1681	1421	41.8	44.1	1.05	1.38
GCA	865	855	598	598	69.1	69.9	1201	1457	47.3	48.2	1.33	1.35
ZZA	1161	869	694	560	59.8	64.5	1626	1458	45.3	42.2	1.55	1.83
NJ11	963	869	694	595	72.1	68.5	1362	1566	44.8	48.8	1.38	1.35
EZ2	963	869	690	606	71.7	69.8	1334	1478	46.2	48.6	1.38	1.35
GC2	1096	1031	736	559	67.2	54.2	1389	1427	45.0	50.2	1.48	1.73
SY63	1237	928	996	697	80.5	75.1	1779	1528	46.8	45.7	1.20	1.50
TQ	1307	869	1016	592	77.8	68.2	1991	2324	45.3	43.8	1.30	1.68
IIY725	1278	979	1025	716	80.2	73.1	1792	2232	45.8	51.1	1.20	1.58
LYPJ	1278	1033	942	764	73.7	74.0	1913	2242	46.0	50.1	1.38	1.53
YLY6	1391	1033	1086	771	78.1	74.7	2031	1875	44.9	46.7	1.28	1.55
HHZ	1096	869	759	562	69.3	64.7	1550	1672	49.0	50.7	1.45	1.85
YLY1	1237	979	839	683	67.9	69.8	1939	2138	47.5	51.3	1.55	1.75
LSD (0.05)	–	–	35	22	2.82	2.33	204	138	2.9	2.3	0.15	0.15

The pre-anthesis RUE was calculated as the ratio of aboveground biomass at heading relative to the intercepted global radiation from transplanting to heading.

**Table 4:** Nitrogen uptake (NUP), nitrogen harvest index (NHI), nitrogen use efficiency for grain production (NUEg) and partial factor productivity of nitrogen fertilizer (PFP) of the varieties grown in different years since 1930s in Middle Reaches of Yangtze River in 2013 and 2014.

Variety	NUP (kg ha^−1^)		NHI (%)		NUEg (kg kg^−1^)		PFP (kg kg^−1^)	
	2013	2014	2013	2014	2013	2014	2013	2014
SLX	138	150	33.9	53.8	29.3	28.8	26.9	28.9
AZZ	172	178	57.3	52.5	36.3	29.8	40.7	35.3
GCA	157	200	52.2	51.7	47.7	34.3	49.7	45.7
ZZA	179	175	56.9	48.8	43.9	40.6	52.0	47.2
NJ11	164	188	55.0	60.4	43.2	37.1	46.7	46.5
EZ2	149	189	56.4	55.6	48.7	36.1	47.3	44.6
GC2	172	168	52.0	58.9	52.3	47.0	59.7	52.1
SY63	186	178	57.1	52.7	46.4	40.3	57.4	47.6
TQ	202	237	57.4	57.9	44.1	29.3	58.9	45.9
IIY725	204	229	54.0	65.0	42.3	39.8	56.3	60.7
LYPJ	189	223	62.2	64.8	50.1	44.5	63.0	65.7
YLY6	208	198	59.3	57.7	46.4	50.5	62.9	66.3
HHZ	157	204	59.4	54.7	58.4	43.4	58.8	58.8
YLY1	206	248	59.1	54.4	46.1	42.0	62.4	69.1
LSD (0.05)	32	20	5.5	4.2	9.1	5.0	4.3	3.6
