# Supplementary material for: Genetic Improvements in Rice Yield and Concomitant Increases in Radiation- and Nitrogen-Use Efficiency in Middle Reaches of Yangtze River
Source: Sci Rep. 2016 Feb 15;6:21049. doi: 10.1038/srep21049 (PMC4753450; doi:10.1038/srep21049)
Supplement: Supplementary Information [file srep21049-s1.doc]

**Genetic Improvements in Rice Yield and Concomitant Increases in Radiation- and Nitrogen- Use Efficiency in Middle Reaches of Yangtze River**

Guanglong Zhu^1^, Shaobing Peng^1^, Jianliang Huang^1^, Kehui Cui^1^, Lixiao Nie^1^, Fei Wang^1^*

Table S1 Growth duration from sowing to heading (HD), HD to maturity (PM) and sowing to PM of the varieties grown in different years since 1930s in Middle Reaches of Yangtze River

| Variety | 2013 | | |  | 2014 | | |  |
| --- | --- | --- | --- | --- | --- | --- | --- | --- |
|  | Sowing-HD | HD-PM | Sowing-PM |  | Sowing-HD | HD-PM | Sowing-PM |  |
| SLX | 77 | 25 | 102 |  | 78 | 29 | 107 |  |
| AZZ | 88 | 36 | 124 |  | 90 | 30 | 120 |  |
| GCA | 75 | 27 | 102 |  | 78 | 29 | 107 |  |
| ZZA | 88 | 42 | 130 |  | 90 | 30 | 120 |  |
| NJ11 | 80 | 37 | 117 |  | 81 | 32 | 113 |  |
| EZ2 | 80 | 37 | 117 |  | 81 | 32 | 113 |  |
| GC2 | 85 | 32 | 117 |  | 85 | 35 | 120 |  |
| SY63 | 93 | 31 | 124 |  | 90 | 30 | 120 |  |
| TQ | 98 | 39 | 137 |  | 85 | 53 | 138 |  |
| ⅡY725 | 95 | 35 | 130 |  | 96 | 37 | 133 |  |
| LYPJ | 95 | 35 | 130 |  | 100 | 33 | 133 |  |
| YLY6 | 102 | 35 | 137 |  | 100 | 38 | 138 |  |
| HHZ | 85 | 32 | 117 |  | 85 | 33 | 118 |  |
| YLY1 | 93 | 31 | 124 |  | 96 | 37 | 133 |  |

Fig. S1 Changes in growth duration and daily grain yield with the year of release of the varieties grown in 2013 (black symbols) and 2014 (gray symbols). Cycles and triangles represent data for inbred and hybrid varieties, respectively.

Fig. S2 Changes in total incident radiation from transplanting to heading stage (a), interception percentage from transplanting to heading stage (b), and intercepted radiation from transplanting to heading stage (c) with the year of release of the varieties grown in 2013 (black symbols) and 2014 (gray symbols). Cycles and triangles represent data for inbred and hybrid varieties, respectively.

Fig. S3 Changes in leaf area index (LAI) and specific leaf weight (SLW, g m^-2^) at heading with the year of release of the varieties grown in 2013 (black symbols) and 2014 (gray symbols). Cycles and triangles represent data for inbred and hybrid varieties, respectively.

Fig. S4 Relationship between grain yield and biomass accumulation, intercepted radiation from transplanting to heading and pre-anthesis RUE in 2013 (black symbols) and 2014 (gray symbols). Cycles and triangles represent data for inbred and hybrid varieties, respectively.

Fig. S5 Relationship between grain yield and nitrogen uptake and nitrogen use efficiency for grain production (NUEg) in 2013 (black symbols) and 2014 (gray symbols). Cycles and triangles represent data for inbred and hybrid varieties, respectively.
